# Supplementary material for: Patterns of Intron Gain and Loss in Fungi
Source: PLoS Biol. 2004 Nov 30;2(12):e422. doi: 10.1371/journal.pbio.0020422 (PMC532390; doi:10.1371/journal.pbio.0020422)
Supplement: Table S1 — Also available at http://genes.mit.edu/NielsenEtAl/. (4.3 MB ZIP). [file pbio.0020422.st001.zip › NielsenEtAl/html/469.html]

AN6732.1.NCU06617.1.MG09470.1.FG05235.1


```
 CLUSTAL W (1.82) Multiple Sequence Alignments - Introns Inserted


Sequence 1: NCU06617.1	147 aa
Sequence 2: MG09470.1	147 aa
Sequence 3: FG05235.1	147 aa
Sequence 4: AN6732.1	132 aa
Alignment Length: 147 aa
Number Identitical Residues: 112 aa
Alignment Score (without introns) 4707


MG09470.1 	MASNHFDSQ~ASTNYKEAFSLFDKRGNGRVALDSLGDLLRACGQNPTLTEIRDLEKNVGG
NCU06617.1	MASNQFDGQ~ASTNYKEAFSLFDKRGNGRVALDSLGDLLRACGQNPTLAEIRELEKNVGG
FG05235.1 	MSNNKFESQ~ASTNYKEAFALFDKRGNGRVTVDSLGDLLRACGQNPTLSEIQDLEKNVGG
AN6732.1  	--------M0ASTNYKEAFSLFDKRGTGRVALESLGDLLRACGQNPTLAEIADLEKNVGG
          	          *********:******.***:::***************:** :*******

MG09470.1 	DF1DFETFQRVLNRPGGFRDPGEPEEYCRGFQVFDKDMTGFIGVGQLKYI~LTNLGEKMT
NCU06617.1	DF1DFETFQRVLNRPGGFRDPGEPEEYCRGFQVFDKDMTGFIGVGQLKYI~LTNLGEKMT
FG05235.1 	EF1DFETFQRVLNRPGGFRDPGEPEEYCRGFQVFDKDMTGFIGVGQLKYI~LTNLGEKMT
AN6732.1  	DF~DFESFLKVLNRPGGFREPGEPEEYCRGFQVFDKDMTGVI-------V1LTNLGEKMT
          	:* ***:* :*********:********************.*       : *********

MG09470.1 	DEEVDELLKSVDTSSGQVNYTE1LVRTILAN
NCU06617.1	DEEVDELLKAVDTSSGQVNYTE1LVRTILAN
FG05235.1 	DEEVDELLKAVDTSSGQVNYTD1LVRTILAN
AN6732.1  	DEEVDELLKAVDTSSGEINYTD1LVRTILAN
          	*********:******::***: ********
```
